# Influence of Visual Stimulation-Induced Passive Reproduction of Motor Images in the Brain on Motor Paralysis After Stroke

**DOI:** 10.3389/fnhum.2021.674139

**Published:** 2021-06-22

**Authors:** Toshiyuki Aoyama, Atsushi Kanazawa, Yutaka Kohno, Shinya Watanabe, Kazuhide Tomita, Fuminari Kaneko

**Affiliations:** ^1^Department of Physical Therapy, Ibaraki Prefectural University of Health Sciences, Ibaraki, Japan; ^2^Department of Physical Therapy, Ibaraki Prefectural University of Health Sciences Hospital, Ibaraki, Japan; ^3^Centre for Medical Sciences, Ibaraki Prefectural University of Health Sciences, Ibaraki, Japan; ^4^Department of Occupational Therapy, Ibaraki Prefectural University of Health Sciences Hospital, Ami, Japan; ^5^Department of Rehabilitation Medicine, Keio University School of Medicine, Shinjuku-ku, Japan

**Keywords:** kinesthetic illusion, visual stimulation, stroke, spasticity, body ownership, mirror therapy

## Abstract

Finger flexor spasticity, which is commonly observed among patients with stroke, disrupts finger extension movement, consequently influencing not only upper limb function in daily life but also the outcomes of upper limb therapeutic exercise. Kinesthetic illusion induced by visual stimulation (KINVIS) has been proposed as a potential treatment for spasticity in patients with stroke. However, it remains unclear whether KINVIS intervention alone could improve finger flexor spasticity and finger extension movements without other intervention modalities. Therefore, the current study investigated the effects of a single KINVIS session on finger flexor spasticity, including its underlying neurophysiological mechanisms, and finger extension movements. To this end, 14 patients who experienced their first episode of stroke participated in this study. A computer screen placed over the patient’s forearm displayed a pre-recorded mirror image video of the patient’s non-paretic hand performing flexion–extension movements during KINVIS. The position and size of the artificial hand were adjusted appropriately to create a perception that the artificial hand was the patient’s own. Before and after the 20-min intervention, Modified Ashworth Scale (MAS) scores and active range of finger extension movements of the paretic hand were determined. Accordingly, MAS scores and active metacarpophalangeal joint extension range of motion improved significantly after the intervention. Moreover, additional experimentation was performed using F-waves on eight patients whose spasticity was reduced by KINVIS to determine whether the same intervention also decreased spinal excitability. Our results showed no change in F-wave amplitude and persistence after the intervention. These results demonstrate the potential clinical significance of KINVIS as a novel intervention for improving finger flexor spasticity and extension movements, one of the most significant impairments among patients with stroke. The decrease in finger flexor spasticity following KINVIS may be attributed to neurophysiological changes not detectable by the F-wave, such as changes in presynaptic inhibition of Ia afferents. Further studies are certainly needed to determine the long-term effects of KINVIS on finger spasticity, as well as the neurophysiological mechanisms explaining the reduction in spasticity.

## Introduction

Stroke, one of the most prevalent neurological diseases worldwide, causes long-term motor impairment. In general, the upper extremities experience greater functional impairment after a stroke compared to the lower extremities, with limited recovery of motor function, especially in the fingers (Langhorne et al., 2009; Houwink et al., 2013). One factor strongly associated with finger motor function is spasticity of the finger flexor muscles (Pundik et al., 2019), which promotes impaired finger extension movements and has a direct negative impact on activities of daily living (ADLs), including eating, grooming, and dressing (Watkins et al., 2002; Sommerfeld et al., 2004). Furthermore, impaired finger extension movement due to spasticity has been assumed to potentially interfere with motor function improvement by increasing the difficultly of therapeutic exercise. Therefore, developing new rehabilitation techniques to reduce finger flexor spasticity may increase the efficiency of therapeutic exercise by improving finger extension movements, thereby contributing to improved performance of ADLs.

Mirror therapy has been one of the proposed treatments for upper limb paralysis after stroke (Altschuler et al., 1999). One feature of mirror therapy is the induction of kinesthetic sensation in the paretic hand by observing the reflected movements of the non-paretic hand in a mirror. A systematic review by Thieme et al. (2018) presented moderate quality evidence showing that mirror therapy promoted better improvement in motor function and motor impairment compared to other interventions. On the other hand, kinesthetic illusion induced by visual stimulation (KINVIS) is a rehabilitation system that can induce a vivid kinesthetic illusion (Kaneko et al., 2007) and can be used together with other intervention modalities (e.g., neuromuscular electrical stimulation) (Kaneko et al., 2019). Given that KINVIS does not require non-paretic hand movement during treatment, the potential for KINVIS to enhance abnormal interhemispheric inhibition associated with non-paretic hand movements is of no concern (Murase et al., 2004; Nowak et al., 2009). Studies in healthy volunteers have shown that motor-related cortical area activation and corticomotor excitability increase during and after KINVIS (Kaneko et al., 2007, 2015, 2016b, 2019; Aoyama et al., 2012; Shibata and Kaneko, 2019). A preliminary study examining the effects of KINVIS among post-stroke patients reported that the single intervention session increased beta band event-related desynchronization obtained from sensorimotor cortex during motor imagery (Okawada et al., 2020), as well as improved paretic upper limb motor function (Kaneko et al., 2016a). Moreover, a study of 11 stroke patients who underwent 10 days of rehabilitation that included KINVIS reported a significant reduction in the spasticity of the finger and wrist flexor muscles and improved upper limb motor function after the intervention (Kaneko et al., 2019). Thus, although KINVIS is expected to be effective in reducing spasticity, it remains unclear whether KINVIS alone is responsible for such an outcome considering that the aforementioned study utilized conventional rehabilitation together with KINVIS. Furthermore, the mechanisms through which KINVIS reduces spasticity have remained unknown. Therefore, Experiment 1 of the current study aimed to determine whether a single session of KINVIS alone could reduce finger flexor spasticity and improve the active range of finger extension in patients with stroke. Moreover, we herein investigated the relationship between changes in spasticity and active range of finger extension, as well as whether subjective illusory sensation and body ownership of the virtual hand presented in the video affected changes in spasticity and active range of finger extension.

Several previous studies using H-reflex and F-wave have shown that patients with stroke exhibiting spasticity have increased spinal reflex excitability (Milanov, 1992a,b; Pisano et al., 2000; Bakheit et al., 2003; Wupuer et al., 2013). In addition, previous studies have shown that F-wave and H-reflex decreases as spasticity is reduced by several interventions (Lo et al., 2009; Kondo et al., 2015; Miyara et al., 2018; Dos Santos et al., 2019). Given the physiological differences between H-reflex and F-wave, both of them assess different aspects of spinal reflex excitability (i.e., the former as a gross measure of alpha motoneuron pool excitability and transmission from the Ia afferent terminals to the alpha motoneurons and the latter as a measure of solely alpha motoneuron excitability) (Milanov, 1992a). However, no study using H-reflex or F-wave has yet investigated whether KINVIS reduces spinal reflex excitability in patients with spasticity. In general, F-wave could be more reliably obtained from the finger muscles compared with H-reflex. Therefore, we determined that F-wave was more suitable than H-reflex for this study, which aimed to identify changes in finger muscle spasticity. Thus, Experiment 2 aimed to elucidate neurophysiological mechanisms explaining the decrease in finger flexor muscle spasticity by recording F-waves from finger muscles to assess the excitability of the alpha motoneuron pool before and after KINVIS.

## Materials and Methods

### Experiment 1

#### Patients

We estimated the sample size by conducting a power analysis using G^∗∗^Power with a power of 0.8, an alpha of 0.05, and an effect size of *d* = 1.0, referring to the effect size obtained in a previous study (Kaneko et al., 2019). A total of 14 (9 men and 5 women; mean age of 61.5 ± 13.4 years) patients who experienced stroke and exhibited spasticity in their paretic finger flexor muscles participated in this experiment, the characteristics of whom are summarized in Table 1. The duration since stroke onset was 16.3 ± 47.1 weeks. The inclusion criterion was patients with stroke over 20 years old who demonstrated finger flexor spasticity [Modified Ashworth Scale (MAS) ≥1]. In addition, previous studies have shown that the presence or absence of kinesthetic sensation has important effects on neurophysiological changes (Kaneko et al., 2007, 2015). Therefore, to investigate the effect of kinesthetic illusion on finger flexor spasticity, rather than just the effect of action observation, patients with subjective illusory sensation or sense of body ownership of ≥1 point on a 7-point Likert scale (see below) were included in this study. Exclusion criteria were as follows: (1) patients with recurrent stroke, (2) with neurological diseases other than stroke, and (3) who did not understand the purpose and task of this study. None of the patients who participated in the study had undergone surgical treatment. One patient (patient no. 7) was taking an anxiolytic drug, alprazolam. Alprazolam also has a muscle relaxant effect; however, this effect is generally weak (Evans, 1981). Patients provided written informed consent prior to study participation in accordance with the Declaration of Helsinki. The present study was approved by the local ethics committee of the Ibaraki Prefectural University of Health Sciences (approval No. e202).

**TABLE 1:** Patient characteristics.

Patient no.	Age	Gender	Lesion side	Diagnosis	FMA upper limb	ARAT	MAS finger flexor muscles	Body ownership	Illusory sensation	Experiment
1	72	W	Lt	Thalamus hemorrhage	7	0	3	2	1	1, 2
2	85	M	Rt	Subcortical infarction	47	41	2	3	2	1
3	73	W	Rt	Putamen and corona radiata infarction	13	0	1	1	2	1, 2
4	51	M	Rt	Putaminal hemorrhage	43	26	2	1	2	1, 2
5	48	M	Rt	Putaminal hemorrhage	26	21	1	1	1	1
6	65	M	Rt	Basal ganglia and corona radiata infarction	41	33	1	3	2	1, 2
7	71	W	Lt	Putaminal hemorrhage	32	16	1	3	0	1, 2
8	72	M	Rt	Pontine infarction	40	20	1	1	1	1
9	44	W	Lt	Pontine hemorrhage	46	35	1	2	2	1, 2
10	55	W	Lt	Subcortical hemorrhage	20	4	1	2	3	1, 2
11	46	M	Lt	Putaminal hemorrhage	40	36	1	2	2	1
12	75	M	Lt	Basal ganglia and corona radiata infarction	9	3	3	3	−1	1
13	45	M	Lt	Putaminal hemorrhage	46	8	3	1	1	1, 2
14	59	M	Lt	Thalamus hemorrhage	29	20	1	2	2	1

*FMA, Fugl-Meyer Assessment; ARAT, Action Research Arm Test; MAS, Modified Ashworth Scale.*

#### Intervention

**FIGURE 1:**
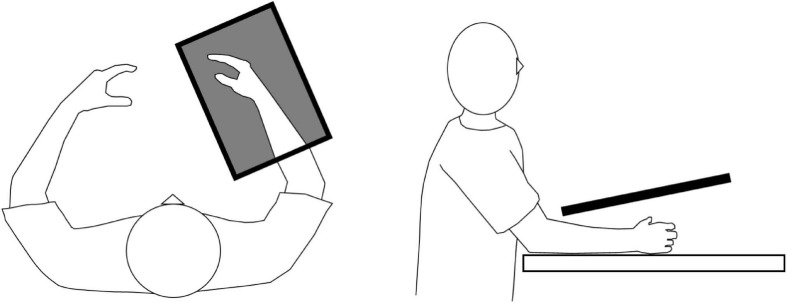
Schematic diagram of the kinesthetic illusion induced by visual stimulation intervention.

All patients underwent a single 20-min session of KINVIS (Aoyama et al., 2020; Okawada et al., 2020). The patients were seated in a comfortable chair with their paretic forearm on the table. Prior to the intervention, the patients were filmed executing the finger flexion–extension movement (3-s flexion and 3-s extension) with the non-paretic hand (Aoyama et al., 2020). During KINVIS intervention (KiNvis Therapy System^TM^; Inter Reha, Tokyo, Japan), the patients were instructed to remain completely relaxed while observing a computer screen projecting a mirror image of the patient’s non-paretic hand placed over their paretic hand (Figure 1). They were instructed to simply observe the movement on the screen, and to not perform motor imagery. The position and size of the artificial hand were adjusted appropriately to create a feeling that the artificial hand belonged to the patient’s own body. KINVIS was performed for 20 min by repeatedly showing the 6-s video of the hand flexion–extension movement.

#### Assessment of Spasticity

Spasticity was assessed using the MAS (Li et al., 2014), which is an ordinal scale with scores of 0, 1, 1+, 2, 3, and 4, and has good or very good intra-rater reliability (Gregson et al., 1999; Ansari et al., 2008). The MAS score for flexor muscles of the index finger was measured during sitting with the forearm in neutral position (Hara et al., 2006) by a single physical therapist with extensive clinical experience with patients with stroke and no conflicts of interest. For statistical analysis, a score of 1 + was transformed to 2, while a score of 2, 3, and 4 was transformed to 3, 4, and 5, respectively (Kaneko et al., 2019).

#### Motor Task and Kinematic Analysis

**FIGURE 2:**
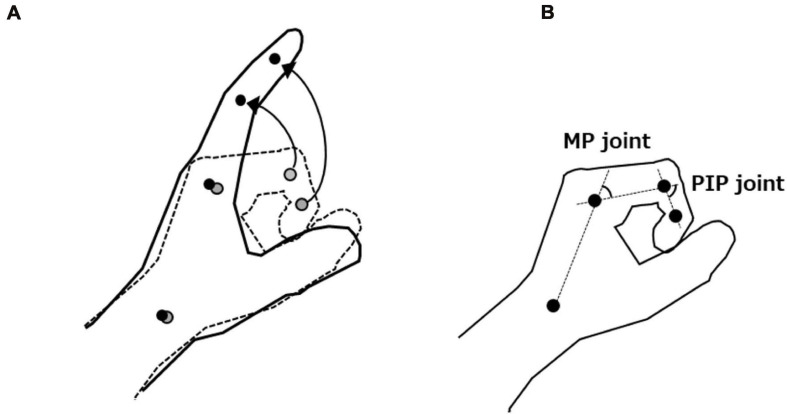
**(A)** Motor task and trajectories of the reflective markers. **(B)** Measurement of flexion angle of the proximal interphalangeal (PIP) and metacarpophalangeal (MP) joints. We defined the active range of PIP and MP joint extension as the difference between each joint’s maximum finger flexion and extension angles.

The patients placed their paretic hand in a neutral position and performed as much finger extension movement as possible from the maximum finger flexion angle for over three times. To focus on finger movements during kinematic analysis, the experimenter fixed the patient’s distal forearm during the motor task. Reflective markers were placed on the landmarks of the radial side of the index finger [distal interphalangeal (DIP), proximal interphalangeal (PIP), and metacarpophalangeal (MP) joints axis] and radial styloid process (Figure 2A). Finger extension movements were captured from above using a digital video camera (EX-100F, 60 frames/s; Casio, Tokyo, Japan). The recorded images were digitized to obtain coordinates for the four reflective markers using a motion analysis system (Frame DIAS V; DKH, Tokyo, Japan). Two-dimensional (2D) coordinates for each marker were run through a fourth-order zero-lag low-pass Butterworth filter (cut-off frequency: 6 Hz). Changes in flexion angle of the PIP and MP joints were calculated from the trajectories of the reflective markers (Figure 2B). For each extension movement, the active range of PIP and MP joint extension from the maximum finger flexion angle was calculated and averaged over three times. PIP joint data in one patient could not be calculated given that the reflective marker of the DIP joint was masked by thumb movement. Such data were therefore excluded from subsequent analysis.

#### Questionnaire Regarding Body Ownership and Illusory Sensation

After KINVIS intervention, patients were asked to rate the sense of body ownership and illusory sensation during intervention using a 7-point Likert scale (–3, strongly disagree; 0, neither agree nor disagree; + 3, strongly agree) (Kaneko et al., 2019; Aoyama et al., 2020).

#### Analysis of Experiment 1

As the obtained data did not show a normal distribution by the Shapiro–Wilk test, the Wilcoxon signed-rank test was performed to determine whether KINVIS promoted changes in hand flexor muscle spasticity and active range of PIP and MP joints extension. The effect size (r) was also calculated by dividing the Z-score derived from each test by the square root of the sample size. Accordingly, effect size was interpreted as small (>0.1), moderate (>0.3), or large (>0.5) based on the guidelines of Cohen (1988). Spearman’s correlation analysis was conducted to determine the relationship between changes in the degree of improvement in finger flexor muscle spasticity (Pre - Post MAS scores, where positive values indicated a decrease in spasticity), the degree of improvement in the active range of finger extension movement (Post - Pre, where positive values indicated an increase in finger extension range of motion), body ownership, and illusory sensation.

### Experiment 2

#### Patients

In order to explore the neurophysiological mechanisms underlying reduced spasticity using KINVIS, the inclusion criteria for Experiment 2 were patients who participated in Experiment 1 and whose MAS scores decreased by at least 1 point after KINVIS intervention. Of the ten participants who met these criteria, two did not agree to participate in the experiment; thus, eight patients (three men and five women; mean age, 59.5 ± 12.2 years) participated in experiment 2. The patients provided written informed consent prior to participation in the experiment in accordance with the Declaration of Helsinki. This experiment was approved by the local ethics committee of the Ibaraki Prefectural University of Health Sciences (approval No. e202).

#### Electromyography

The skin area of the electrode attachment was swabbed with alcohol and prepared using an abrasive skin-prepping gel, after which surface Ag–AgCl electrodes were placed over the bilateral first dorsal interosseous (FDI). Electromyography (EMG) signals were amplified (Neuropack MEB2300; Nihon Kohden, Saitama, Japan) at a gain of 0.2–0.5 mV per division and band-pass filtered at 5–5 kHz. All signals were stored on a computer for offline analysis. The sampling frequency was set at 10 kHz.

#### F-wave

The patient’s arm was placed on a table, and the elbow was flexed to approximately 90°, with the forearm in a supinated position. F-waves were recorded from the affected and non-affected FDI muscle, which is involved in index finger flexion. In addition, the F-wave amplitude and persistence of the paretic FDI muscle in patients with stroke with spasticity have been shown to be significantly increased as compared with those of the FDI muscle in healthy subjects (Wupuer et al., 2013). For these reasons, we chose FDI as the target muscle for the F-wave. Supramaximal electrical stimulation was applied to the ulnar nerve at the wrist using a 0.2-ms rectangular electrical pulse (Aoyama et al., 2019). At least 30 F-waves were recorded under resting conditions. When a visually evident involuntary contraction of the FDI muscle was observed, the trial was rejected and another trial was recorded. F-wave persistence was defined as the ratio of trials in which F-wave amplitudes greater than 50 μV were obtained to the total number of trials. The F/M amplitude was defined as the ratio of the F-wave amplitude to the maximum M-wave amplitude.

#### Analysis of Experiment 2

One patient (patient no. 13) was having difficulty in holding the test arm position due to the strong spasticity of the forearm flexor and pronator muscles. Owing to this, we had difficulty fixing the stimulating electrode to the ulnar nerve for this patient. As a result, stable M-waves could not be obtained. Therefore, this patient’s data were excluded from further analysis. To test the normality of the data, we performed the Shapiro–Wilk test. Since F/M amplitudes showed strongly positive skewed distributions and normality could not be obtained, logarithmic transformation was performed (Osborne, 2002; Bland et al., 2013). After the logarithmic transformation, kurtosis and skewness approached zero, and the Shapiro–Wilk test showed a normal distribution. The effects of time (pre- and post-intervention) and hand (paretic and non-paretic hands) factors on M-wave amplitude, F-wave persistence, and F/M amplitude were determined using two-way repeated measures analysis of variance. Partial η^2^ was calculated as a measure of effect size (small: 0.01; medium: 0.06; large: 0.14) (Huck, 2011).

## Results

### Experiment 1

#### Spasticity

Spasticity assessed via MAS was significantly reduced after a single session of KINVIS (*Z* = 2.972, *n* = 14, *p* = 0.003, effect size *r* = 0.794; Table 2). Among the 14 patients included herein, 10 showed at least a 1-point decrease in the MAS score, whereas none of the patients showed worsening symptoms.

**TABLE 2:** Results of finger spasticity and active range of finger extension.

	Pre-intervention	Post-intervention	*p* value
**Finger flexor spasticity:**			
MAS score (0–5): Median (first quartile, third quartile)	1 (1, 3)	1 (0, 1)	0.003*
MAS score 0: Number of subjects	0	5	
MAS score 1: Number of subjects	9	8	
MAS score 2: Number of subjects	2	1	
MAS score 3: Number of subjects	3	0	
**Active range of finger extension:**			
MP joint: Median (first quartile, third quartile)	23.6 (7.8, 51.5)	27.8 (10.7, 47.9)	0.048*
PIP joint: Median (first quartile, third quartile)	68.2 (24.1, 89.6)	64.6 (33.5, 82.8)	0.507

MAS, Modified Ashworth Scale; MP, metacarpophalangeal; PIP, proximal interphalangeal. For statistical analysis, MAS scores of 1 + and 2 are presented as 2 and 3, respectively. * indicates significant difference (*p* < 0.05).

#### Active Range of Proximal Interphalangeal and Metacarpophalangeal Joint Extension

No significant difference in the active range of PIP joint extension was observed before and after the intervention (*Z* = 0.664, *n* = 13, *p* = 0.507, effect size *r* = 0.184; Table 2). However, the active range of MP extension was significantly increased after KINVIS (*Z* = 1.977, *n* = 14, *p* = 0.048, effect size *r* = 0.528).

#### Relationship Between Finger Flexor Spasticity, Active Range of Finger Extension, Body Ownership, and Illusory Sensation

Spearman’s rank correlation test showed no significant correlation between improvement in MAS score of the finger flexor muscle, improvement in active range of PIP and MP joint extension, body ownership, and illusory sensation in the artificial hand (improvement in MAS score vs. improvement in active range of PIP extension: rs = 0.178, *n* = 13, *p* = 0.543; improvement in MAS score vs. improvement in active range of MP extension: rs = –0.366, *n* = 14, *p* = 0.199; improvement in MAS score vs. body ownership: rs = 0.395, *n* = 14, *p* = 0.162; improvement in MAS score vs. illusory sensation: rs = –0.222, *n* = 14, *p* = 0.446, improvement in active range of PIP extension vs. body ownership: rs = –0.096, *n* = 13, *p* = 0.745; improvement in active range of PIP extension vs. illusory sensation: rs = 0.188, *n* = 13, *p* = 0.520; improvement in active range of MP extension vs. body ownership: rs = –0.464, *n* = 14, *p* = 0.095; improvement in active range of MP extension vs. illusory sensation: rs = –0.069, *n* = 14, *p* = 0.815).

### Experiment 2

**FIGURE 3:**
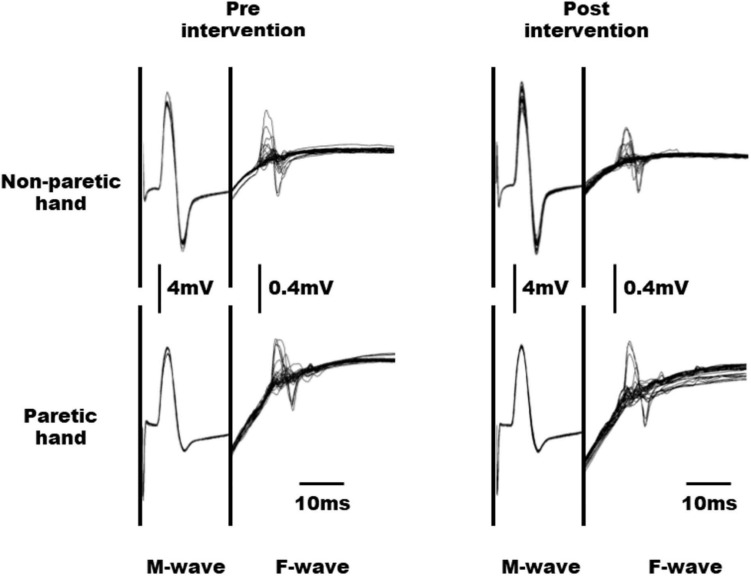
Raw waveforms of M and F waves obtained from the non-paretic and paretic hands in a representative subject before and after intervention.

The raw waveforms of M and F waves obtained from the paretic and non-paretic hands of a representative subject before and after the intervention are shown in Figure 3. No significant interaction was observed between time and hand factors in the M-wave amplitude (F1, 6 = 0.324, *n* = 7, *p* = 0.590, effect size partial η^2^ = 0.051; Table 3). Moreover, both time (F1, 6 = 0.482, *n* = 7, *p* = 0.514, effect size partial η^2^ = 0.074) and hand factors (F1, 6 = 3.441, *n* = 7, *p* = 0.113, effect size partial η^2^ = 0.365) showed no significant main effect on the M-wave amplitude. For the F/M amplitude, no significant interaction was obtained between time and hand factors (F1, 6 = 0.356, *n* = 7, *p* = 0.572, effect size partial η^2^ = 0.056). The paretic hand had a significantly larger F/M amplitude than the non-paretic hand (F1, 6 = 10.704, *n* = 7, *p* = 0.017, effect size partial η^2^ = 0.641). The time factor had no significant main effect on the F/M amplitude (F1, 6 = 0.115, *n* = 7, *p* = 0.747, effect size partial η^2^ = 0.019). No significant interaction was observed between the time and hand factors on F-wave persistence (F1, 6 = 1.723, *n* = 7, *p* = 0.237, effect size partial η^2^ = 0.223). Although the *p* value for the hand factor did not reach significance, the paretic hand tended to have higher F-wave persistence than the non-paretic hand (F1, 6 = 4.200, *n* = 7, *p* = 0.086, effect size partial η^2^ = 0.412). The time factor showed no significant main effect on F-wave persistence (F1, 6 = 0.003, *n* = 7, *p* = 0.957, effect size partial η^2^ = 0.001).

**TABLE 3:** Results of M-wave, F-wave persistence and F/M amplitude.

		Pre-intervention	Post-intervention	*p* value		
				Interaction	Main effect	
					Hand factor	Time factor
M-wave amplitude (mV)	Non-paretic hand	11.7 (4.1)	11.1 (3.4)	0.590	0.113	0.514
	Paretic hand	8.0 (4.6)	7.9 (4.3)			
F-wave persistence (%)	Non-paretic hand	69.1 (24.4)	63.3 (20.5)	0.236	0.086	0.957
	Paretic hand	86.0 (16.7)	91.4 (10.2)			
F/M amplitude (%)	Non-paretic hand	1.31 (1.15)	1.18 (1.09)	0.572	0.017*	0.747
	Paretic hand	2.61 (1.87)	2.61 (1.48)			

Data are presented as mean (standard deviation). * indicates significant difference (*p* < 0.05).

## Discussion

The current study showed that a single 20-min session of KINVIS immediately reduced MAS score and increased the active range of MP joint extension, suggesting its potential utility in improving finger flexor spasticity and finger extension movements in patients with stroke for whom effective treatments are limited.

After 1 session of 20-min KINVIS intervention, MAS score decreased significantly with a large effect size, while 71.4% of the patients exhibited a 1 point and greater decrease in MAS score. Chen et al. (2019) reported that the minimal clinically important difference (MCID) in upper limb spasticity using MAS scores in patients with stroke was either 0.48 (medium effect size) or 0.76 (large effect size). This indicates that KINVIS promoted an improvement even greater than the MCID in over 70% of the patients, suggesting that KINVIS may be one of the clinically meaningful interventions for finger flexor spasticity in patients with stroke. On the other hand, mirror therapy, a technique that induces a kinesthetic illusion similar to KINVIS, has long been used to treat paretic upper limb in patients with stroke (Perez-Cruzado et al., 2017). However, mirror therapy has generally been considered to have no effect on upper limb spasticity (Yavuzer et al., 2008; Samuelkamaleshkumar et al., 2014; Perez-Cruzado et al., 2017). Although our results cannot elucidate why KINVIS and mirror therapy have different effects on spasticity, we surmise that differences in the nature of both interventions are involved. In particular, the crucial difference between both interventions is presence of non-paretic hand movement. The subjective kinesthetic sensation induced during mirror illusion is markedly affected by proprioceptive afferent input from the non-paretic hand (Chancel et al., 2016). Furthermore, the non-paretic hand movements may reinforce abnormal interhemispheric inhibition (Murase et al., 2004; Nowak et al., 2009). Conversely, the aforementioned effects caused by non-paretic hand movements certainly do not occur during KINVIS because of the use of a pre-recorded mirror image video. Indeed, Kaneko et al. (2019) reported that a 10-day intervention, including KINVIS, significantly reduced upper limb spasticity. However, given that their study concurrently applied KINVIS and NMES while also including conventional therapeutic exercise in the overall intervention protocol, whether KINVIS directly contributed to the reduction in upper limb spasticity remains unclear. Other effective treatments for spasticity include botulinum toxin injection (Brashear et al., 2002; Ro et al., 2020) and acupuncture (Tavakol et al., 2020). However, because these treatments are invasive, KINVIS is expected to be beneficial as a non-invasive treatment for spasticity. Further studies are needed to compare the efficacy and cost of KINVIS with those of other therapies for clinical application.

Our results showed that KINVIS intervention acutely increased the active range of MP joint extension. Studies have shown that decreased finger extension is one of the most common deficits in patients with stroke (Kamper and Rymer, 2001; Raghavan, 2007). The ability to extend the fingers by at least 10° has been one of the general inclusion criteria for constraint-induced movement therapy, which has been proven highly effective in treating motor dysfunction among patients with upper limb paralysis (Lin et al., 2019). Moreover, reports have shown that the ability to voluntarily extend the fingers was closely associated with the effects of constraint-induced movement therapy (Fritz et al., 2005). Therefore, the present results showing improved range of finger extension after the intervention suggests the potential utility of KINVIS in improving finger extension function to an extent where task-oriented upper limb training can be performed or as a conditioning intervention that aids in the effective performance of such training.

No significant correlation had been noted between the degree of improvement in spasticity and the degree of increase in active finger extension range of MP and PIP joint motion. This result implies that changes in these variables were independent of each other. A case report of a patient with stroke showed increased extensor digitorum activity after a single session of KINVIS (Aoyama et al., 2020). Therefore, change in hand extensor muscle activity may be a candidate factor affecting the improvement in active range of finger extension apart from hand flexor muscle spasticity. Excessive muscle contraction of the finger flexor muscle during active finger extension movement may be another factor contributing to improved range of finger extension (Kamper et al., 2003). As such, clarifying the factors that contribute to improved active range of finger extension by examining the changes in finger extensor and flexor activities during active finger extension movement is certainly necessary. The sense of body ownership and illusory sensation was not significantly correlated with the degree of improvement in spasticity and active range of finger extension. Notably, a previous study showed that the intensity of illusory sensation positively correlated with changes in corticomotor excitability, but it was not statistically significant (Aoyama et al., 2012). Therefore, the degree of illusory sensation and body ownership may not necessarily exert a strong influence on the improvement of spasticity and motor function. However, because this study did not include patients who did not experience any illusory sensation and sense of body ownership, we cannot rule out that these are not related to symptom improvement.

Our finding showed that the paretic FDI muscle had a significantly higher F/M amplitude compared to the non-paretic FDI muscle. Moreover, the paretic FDI muscle tended had higher F-wave persistence than the non-paretic FDI muscle. These results are consistent with those presented in a previous study that examined F-waves in patients with stroke showing spasticity (Milanov, 1992a,b; Wupuer et al., 2013). Thus, the subjects included herein had increased spinal excitability before the intervention, supporting the presence of spasticity. On the other hand, despite the significant decrease in MAS score after KINVIS, no significant changes in F-wave amplitude and persistence were noted. Given that F-waves are generated by the backfiring of antidromically activated motoneurons (Mcneil et al., 2013), they are solely affected by alpha motoneuron excitability. Conversely, F-waves are not affected by the presynaptic inhibition of Ia afferent terminals, unlike H-waves, which are produced by Ia afferent firing (Pierrot-Deseilligny, 1997). Therefore, the absence of changes in the F-wave after KINVIS, despite the reduction in spasticity, may be due to the physiological changes that could not be detected by the F-wave, such as changes in presynaptic inhibition of Ia afferent input or reciprocal Ia inhibition. The Ia reciprocal and Ia presynaptic inhibitory interneurons receive descending drive (Jankowska and Tanaka, 1974; Cowan et al., 1986; Rothwell et al., 1991; Meunier and Pierrot-Deseilligny, 1998). Furthermore, the excitability of these inhibitory interneurons has been shown to be modulated before the onset of EMG activity of the antagonist muscle (Tanaka, 1976; Nielsen and Kagamihara, 1993). We speculate that KINVIS may selectively modulate the excitability of these inhibitory interneurons, without producing muscle activity. Supporting this hypothesis, Kawakami et al. (2018) reported that motor imagery enhanced the presynaptic inhibition of Ia afferent input and disynaptic reciprocal Ia inhibition of antagonists in patients with stroke. One study suggested that KINVIS should be interpreted as implicit motor imagery (Hanakawa, 2016) wherein the movement observed by the subjects in the video during KINVIS is passively imagined. In support of this notion, functional magnetic resonance imaging studies have shown that brain network activity detected during KINVIS was similar to that during motor imagery (Kaneko et al., 2015). Therefore, the findings of Kawakami et al. support our aforementioned assumptions. Future studies will need to examine the neurophysiological mechanisms of spasticity reduction following KINVIS using H-reflex and H-reflex conditioning-test paradigm. Moreover, there is a need to examine differences in neurophysiological changes between patients whose MAS scores decreased, or did not.

One of the most important limitations of this study is the absence of control groups or conditions. Therefore, we cannot deny the possibility that the results obtained in this study were due to the maintenance of rest or action observation. It is necessary to compare the effects of the KINVIS intervention with control tasks, such as rest or action observation, and with control patients who do not experience a sense of body ownership or illusory sensation. Furthermore, in the present study, patients who subjectively experienced a certain level of kinesthetic illusory sensation or a sense of body ownership were included to investigate the effects of kinesthetic illusions rather than the effects of mere action observation. Therefore, whether patients who do not experience kinesthetic illusory sensations or a sense of body ownership would experience improvements in the finger flexor spasticity and finger extension movement remains unclear. This issue should be examined in the future study.

In conclusion, the present study investigated the effects of a single KINVIS session on finger flexor spasticity, including its underlying neurophysiological mechanisms, and finger extension movements. Accordingly, our results showed that KINVIS significantly improved the MAS score and active range of MP joint extension. Moreover, no changes in F-wave persistence and amplitude had been noted after the intervention. The aforementioned results suggest that KINVIS may have clinical significance as a novel intervention for improving finger flexor spasticity and finger extension movements even when applied without NMES in patients with stroke. Given that the F-wave used herein could not identify the mechanism through which KINVIS reduces spasticity, future studies using H-reflex and/or H-reflex conditioning-test paradigm are warranted.

## Data Availability Statement

The raw data supporting the conclusions of this article will be made available by the authors, without undue reservation.

## Ethics Statement

The studies involving human participants were reviewed and approved by the Local Ethics Committee of the Ibaraki Prefectural University of Health Sciences. The patients/participants provided their written informed consent to participate in this study. Written informed consent was obtained from the individual(s) for the publication of any potentially identifiable images or data included in this article.

## Author Contributions

TA and FK designed the study. YK and SW contributed to the clinical assessment. TA, AK, YK, KT, and FK collected the kinematic and neurophysiological data. TA, AK, and FK contributed to data analysis and interpretation. All authors contributed to wrote the manuscript, read, and approved the final manuscript.

## Conflict of Interest

FK is the founding scientist of INTEP Inc., a commercial company for the development of rehabilitation devices since July 2019. This company does not have any relationship with the device or setup used in the present study. FK received license fees from Inter Reha Co., Ltd. The remaining authors declare that the research was conducted in the absence of any commercial or financial relationships that could be construed as a potential conflict of interest.
